# Anti-scarring effects of conbercept on human Tenon’s fibroblasts: comparisons with bevacizumab

**DOI:** 10.1186/s12886-023-02914-4

**Published:** 2023-04-26

**Authors:** Lei Zuo, Shaopin Zhu, Shengjie Gu, Xun Xu

**Affiliations:** 1 Department of Ophthalmology, Shanghai Fourth People’s Hospital, Tongji University School of Medicine, No. 1279, Sanmen road, Shanghai, 200434 China; 2 Department of Ophthalmology, Shanghai General Hospital, Shanghai Jiao Tong University School of Medicine, No. 85 / 86, Wujin road, Shanghai, 200080 China

**Keywords:** Anti-scarring, Bevacizumab, Conbercept, glaucoma filtration surgery, Human Tenon’s fibroblasts, Placental growth factor, Vascular endothelial growth factor

## Abstract

**Background:**

Safely inhibiting the formation of scar in the glaucoma filtration surgery (GFS) has always been an issue for clinical glaucoma doctors. Anti-vascular endothelial growth factor (VEGF) agents can reduce angiogenesis, and anti-placental growth factor (PIGF) agents can affect reactive gliosis. However, the effect of conbercept, which can bind to both VEGF and PIGF, on human Tenon’s fibroblasts (HTFs) is unknown.

**Methods:**

HTFs were cultured in vitro and treated with conbercept or bevacizumab (BVZ). No drug was added to the control group. The effects of drugs on cell proliferation were assessed using the 3-(4,5-dimethylthiazol-2-yl)-2,5-diphenyltetrazolium bromide (MTT) assay, and the collagen type I alpha1(Col1A1) mRNA expression level was measured using quantitative polymerase chain reaction (qPCR). HTF cell migration after drug interventions was evaluated using the scratch wound assay along with the measurement of the expression levels of VEGF and PIGF in human umbilical vein endothelial cells (HUVECs) using enzyme-linked immunosorbent assay, as well as the detection of the VEGF(R) mRNA expression level in HTFs using qPCR.

**Results:**

After the addition of conbercept (0.01, 0.1, and 1 mg/mL) to the cultured HTFs or HUVECs, no significant cytotoxicity was observed compared with the control group, while the cytotoxicity of 2.5 mg/mL BVZ on HTFs was obvious. Conbercept significantly inhibited HTF cell migration and Col1A1 mRNA expression level in HTFs. It was superior to BVZ in inhibiting HTF migration. After the intervention with conbercept, the expression level of PIGF and VEGF in HUVECs significantly decreased; and the inhibitory effect of conbercept on the expression level of VEGF in HUVECs was weaker than that of BVZ. Conbercept was more advantageous than BVZ in inhibiting the expression level of VEGFR-1 mRNA in HTFs. However, its effect in terms of inhibiting the expression level of VEGFR-2 mRNA in HTFs was weaker than that of BVZ.

**Conclusion:**

The results suggested the low cytotoxicity and significant anti-scarring effect of conbercept in HTF with significant anti-PIGF and inferior anti-VEGF effects compared with BVZ, thus providing a better understanding of the role of conbercept in the GFS wound healing process.

## Background

Surgery must be performed in cases where maximal medical therapy cannot control intraocular pressure (IOP) in patients with glaucoma. Glaucoma filtration surgery (GFS) is currently one of the most effective methods for treating glaucoma [1, 2]. The goal of GFS is to create an incision to bypass the trabecular meshwork and drain the aqueous humor outward through the subconjunctival filtering bleb to relieve the elevated IOP [3]. Unlike with most surgeries, the success of GFS is achieved by inhibiting wound healing [4]. Postoperative conjunctival scarring at the site of the filtering bleb, however, promotes adhesion to the episcleral tissue, which leads to the resealing of the bleb inhibiting the aqueous flow and poor control of IOP [5]. Human Tenon’s fibroblast (HTF) is regarded as the major cell type contributing to the formation of subconjunctival scar after GFS [6].

Safely inhibiting the formation of scars in GFS and improving the success rate of surgery has noticeably attracted the attention of glaucoma specialists. Antimetabolites, such as 5-fluorouracil (5-Fu) and mitomycin-C (MMC), are used to modulate the healing process and improve the success rate of surgery. However, despite their effectiveness, these drugs can lead to thin-walled filtering bleb, which is related to the high-risk of leakage, hypotony, and endophthalmitis [7, 8]. When vascular endothelium growth factor (VEGF) expression is upregulated in the early stage of GFS [9, 10], the treated eyes receiving subconjunctival injection of bevacizumab (BVZ) can develop larger filtration blebs than the non-treated eyes [11], or the IOP is reduced with a better safety profile compared with the MMC-treated group [12]. However, the scar formation after GFS involves complex processes of angiogenesis and fibrosis, and hence it is inadequate to aim only at anti-VEGF or other single targets [13–15].

Placental growth factor (PIGF) is primarily a pro-angiogenic growth factor only upregulated under pathological conditions [16]. Previous studies [17] showed that the inhibition of PIGF could effectively reduce angiogenesis, vascular leakage, and inflammation, besides affecting reactive gliosis in the retina. Conbercept can bind to dual targets (VEGF and PIGF) for antiangiogenic therapy [18–20]. Zhang et al. [21] used the subconjunctival injection of conbercept as an adjuvant to GFS for open-angle glaucoma and compared its efficacy with that of 5-Fu. Less vascularity of filtration blebs, lower IOP, and lower incidence of corneal epithelial stripping were achieved after the surgery in the conbercept treatment group. However, evidence showing the direct effects of conbercept on HTFs [22, 23] and its safety profile is still lacking. Also, the underlying mechanisms of conbercept in inhibiting scar formation in GFS are still unclear.

In the present study, HTFs and HUVECs were cultured in vitro and then treated with conbercept, BVZ, 5-Fu, or MMC. The results revealed that conbercept significantly inhibited HTF cell migration and collagen type I alpha1 (Col1A1) mRNA expression level [24] in HTFs with a significant anti-PIGF effect and an inferior anti-VEGF effect compared with BVZ; also, the low cytotoxicity of conbercept was observed. Our research might assist in better understanding the role of conbercept during the GFS wound healing process.

## Materials and methods

### Cell culture, drugs, and reagents

Following the Declaration of Helsinki, HTFs were obtained from the specimens by excising the Tenon’s capsule during strabismus surgery [23]. The study was approved by the ethics committee of Shanghai Fourth People’s Hospital Affiliated to Tongji University School of Medicine (Approval No. 2,019,012). HTFs were cultured in Dulbecco’s modified Eagle’s medium (DMEM) supplemented with 10% fetal bovine serum (FBS) and antibiotics. HUVECs (CRL-2873; American Type Culture Collection) were cultured in a DMEM/F12 medium containing 10% FBS and antibiotics. The medium, antibiotics, trypsin (1:250), recombinant human VEGF, 3-(4,5-dimethylthiazol-2-yl)-2,5-diphenyltetrazolium bromide (MTT), and heat-inactivated FBS were purchased from Invitrogen (CA, USA). An endothelial cell culture medium was obtained from PromoCell GmbH (Heidelberg, Germany). Conbercept (10 mg/mL) was purchased from Chengdu Kanghong Biotechnologies Co. Ltd. (Chengdu, China). Bevacizumab (Avastin) (25 mg/mL), PhosSTOP, and protease inhibitors were obtained from Roche (Basel, Switzerland). An enzyme-linked immunosorbent assay (ELISA) kit was purchased from R&D Systems (Minneapolis, Minnesota, USA). A Bradford protein assay kit was obtained from Bio-Rad Laboratories Inc. (Hercules, California, USA). 5-Fu was provided by Shanghai Xudong Haipu Pharmaceutical Co., Ltd. (Shanghai, China), and MMC was purchased from Zhejiang Hisun Pharmaceutical Co., Ltd. (Zhejiang, China). Phosphate-buffered saline (PBS) and 0.9% sodium chloride were provided by Baxter Medical Products Co., Ltd. (IL, USA).

### MTT assay for the cytotoxicity/proliferation of HTFs and HUVECs

A single-cell suspension cultured under normal conditions in the logarithmic growth phase was inoculated into six-well culture plates at a density of 5 × 10^4^ cells per well and synchronized with a serum-free Roswell Park Memorial Institute (RPMI)-1640 medium. HTFs were incubated with conbercept (0.01, 0.1, and 1 mg/mL), BVZ (0.025, 0.25, and 2.5 mg/mL), 5-Fu (0.05, 0.5, and 5 mg/mL), MMC (0.0002, 0.002, and 0.02 mg/mL), conbercept (0.1 mg/mL) + 5-Fu (0.05, 0.5, and 5 mg/mL), Conbercept (0.1 mg/mL) + MMC (0.0002, 0.002, and 0.02 mg/mL), or PBS (control) for 24 h. Meanwhile, HUVECs received the same treatments as HTFs for 24 h. The cells were rinsed with PBS, and then a fresh serum-free medium with or without 0.5 mg/mL MTT was added to the cells. After 2 h of incubation, the formazan extraction amount and the absorbance value were measured using an ELISA kit (Emax; Molecular Devices Corp., CA, USA) at 570 nm [25, 26].

### Analysis of HTF cell migration

When HTFs reached a confluence of 80% in vitro, scratches were drawn vertically to a pre-drawn line with a 1-mm tip at the bottom of the culture dish, and three scratches were drawn at the same distance. The cells floating along the scratches were washed with PBS and photographed under a microscope (DM IRB, Leica, Wetzlar, Germany; magnification, 40×). The time point was recorded as 0 h, in which six images at different fields of view were taken. This was followed by the addition of 0.2% FBS and treatment of cells with conbercept (0.1 mg/mL), BVZ (0.25 mg/mL), 5-Fu (0.5 mg/mL), MMC (0.002 mg/mL), conbercept (0.1 mg/mL) + 5-Fu (0.5 mg/mL), or conbercept (0.1 mg/mL) + MMC (0.002 mg/mL). Then, 30 ng/mL VEGF was added to the treatments, while 30 ng/mL VEGF alone was added to a 0.2% FBS culture medium, which acted as the control group. The cells were further cultured, and the images were taken after culturing for 12 h. The same region was selected for each repetition when taking the photographs. The area of the wound was determined with Image J (v1.41, NIH, MD, USA). The wound closure rate was calculated using the following formula: wound closure rate = (area of the wound at 0 h – area of the wound at12 h)/area of the wound after 0 h [23, 27].

### Quantitative polymerase chain reaction (qPCR) analysis of Col1A1 mRNA and VEGF(R) mRNA in HTFs

HTFs were cultured in vitro, and conbercept (0.1 mg/mL), BVZ (0.25 mg/mL), 5-Fu (0.5 mg/mL), MMC (0.002 mg/mL), conbercept (0.1 mg/mL) + 5-Fu (0.5 mg/mL), conbercept (0.1 mg/mL) + MMC (0.002 mg/mL), or PBS (control) was added, respectively; after 24 h, the expression level of Col1A1 mRNA and VEGF(R) mRNA was quantitatively analyzed using qPCR [23, 24] (n = 3). The total mRNA was extracted by using TRIzol reagent (Invitrogen, Carlsbad, CA). cDNA was then synthesized by reverse transcription (Tetro cDNA Synthesis Kit, Bioline, London, UK), and mRNA was detected by RT-PCR (SensiFAST^TM^SYBR® Hi-ROX Kit, Bioline, London, UK) by using a special software (ABI Prism 7500 SDS Software, USA). The designed primer sequences [23, 24] are shown in Table 1. The expression level of the expression levels of VEGF, VEGFR-1 (Flt-1), VEGFR-2 (KDR), and Col1A1 mRNA was normalized to the expression level of glyceraldehyde-3-phosphate dehydrogenase (GAPDH) mRNA.

**Table 1:** Primers used in real-time polymerase chain reaction

Gene name	Primer sequences
Col1A1	Forward: 5ʹ-AAAGATGGACTCAACGGTCTC-3ʹ
	Reverse: 5ʹ-CATCGTGAGCCTTCTCTTGAG-3ʹ
VEGF	Forward: 5ʹ-ATCGAGTACATCTTCAAGCCAT-3ʹ
	Reverse: 5ʹ-GTGAGGTTTGATCCGCATAATC-3ʹ
Flt-1	Forward: 5ʹ-CAAGATTTGCAGAACTTGTGGA-3ʹ
	Reverse: 5ʹ-CTGTCAGTATGGCATTGATTGG-3ʹ
KDR	Forward: 5ʹ-GGAGCTTAAGAATGCATCCTTG-3ʹ
	Reverse: 5ʹ-GATGCTTTCCCCAATACTTGTC-3ʹ
GAPDH	Forward: 5ʹ-AGACAGCCGCATCTTCTTGT-3ʹ
	Reverse: 5ʹ-CTTGCCGTGGGTAGAGTCAT-3ʹ

*Note.* Col1A1: collagen type I alpha1; VEGF: vascular endothelial growth factor; Flt-1: VEGFR-1; KDR: VEGFR-2; GAPDH: glyceraldehyde-3-phosphate dehydrogenase

### Detection of the expression levels of VEGF and PIGF in HUVECs using ELISA

HUVECs were cultured in vitro and treated with conbercept (0.1 mg/mL), BVZ (0.25 mg/mL), 5-Fu (0.5 mg/mL), MMC (0.002 mg/mL), conbercept (0.1 mg/mL) + 5-Fu (0.5 mg/mL), conbercept (0.1 mg/mL) + MMC (0.002 mg/mL), or PBS (control). After 24 h, 200 µL of the supernatant per well was collected and analyzed with a VEGF-ELISA kit (R&D Systems, USA) and a PIGF-ELISA kit (R&D Systems, USA) following the manufacturer’s protocols [28, 29].

### Statistical analysis

Variables were described as mean ± standard deviation. When variances were homogeneous, the least significant difference and the Student–Newman–Keuls (SNK) tests were used to analyze variances. When the differences were inhomogeneous, the rank-sum test was used to analyze the differences between the experimental groups. The statistical analysis was performed using SPSS 19.0 (IBM, NY, USA). A *P* value < 0.05 indicated a statistically significant difference.

## Results

### Low cytotoxicity of the drugs

**Fig. 1:**
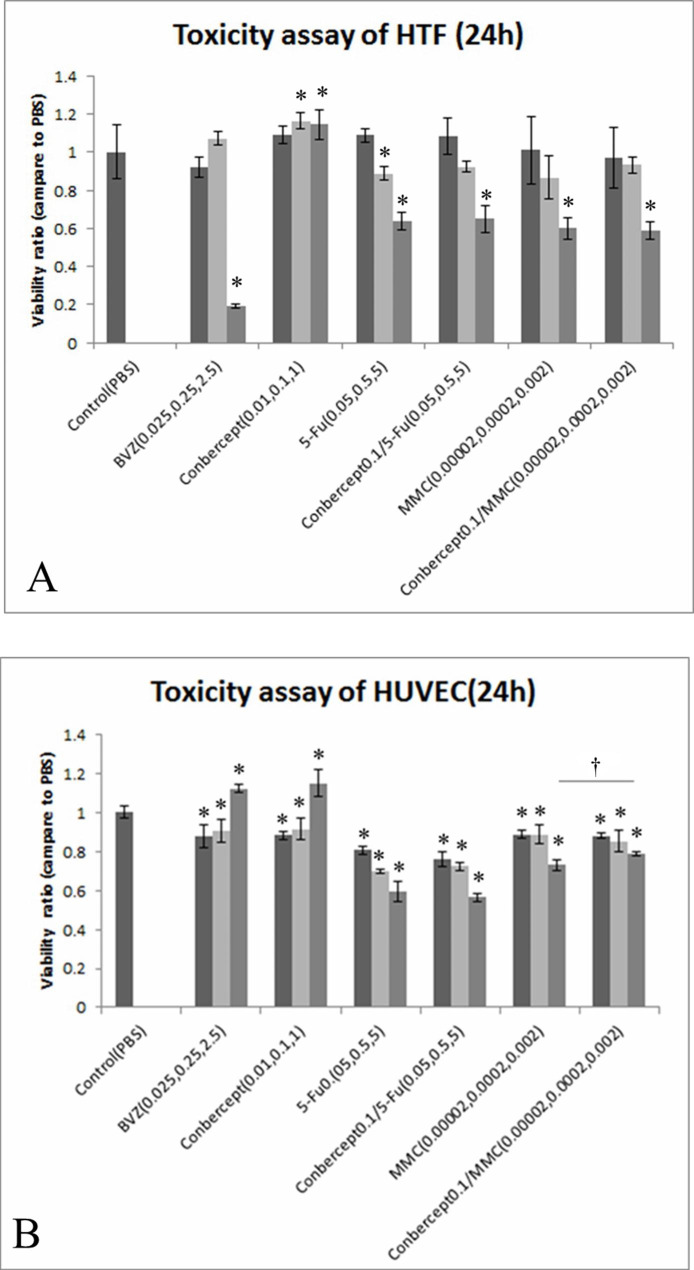
Viability of (A) human Tenon’s fibroblasts (HTFs) and (B) human umbilical vein endothelial cells (HUVECs) after treatment with conbercept, bevacizumab (BVZ), mitomycin C (MMC), 5-fluorouracil (5-Fu), conbercept / 5-Fu, and conbercept / MMC. The cell viability in the control group was set to 100%. Unit: mg/ml; *P < 0.05

After the addition of conbercept (0.01, 0.1, and 1 mg/mL) to the cultured HTFs or HUVECs, no significant cytotoxicity was observed compared with that in the control group, and the cytotoxicity did not increase with the elevation of the drug concentration. The cytotoxicity of BVZ (2.5 mg/mL) in HTFs was more obvious compared with that in the control group (*P* < 0.05). The cytotoxicity of conbercept + 5-Fu in HTFs and HUVECs was the same as that of 5-Fu; the cytotoxicity of conbercept + MMC in HTFs was not higher than that of MMC. In HUVECs, the cytotoxicity of conbercept (0.1 mg/mL) + MMC (0.002 mg/mL) was lower than that of MMC (0.002 mg/mL) (*P* < 0.05) (Tables 2 and 3; Fig. 1).

**Table 2:** Cell viability of human Tenon fibroblasts (HTFs) after treatment with medicines

Medicine and concentration (mg/ml)		Absorbance at 570 nm (ratio, vs. PBS)	P (vs. control)	Medicine and concentration (mg/ml)		Absorbance at 570 nm (ratio, vs. PBS)	P (vs. control)	P
Control (PBS)		1.0000 ± 0.1429						
BVZ	0.025	0.9205 ± 0.0546	0.128					
	0.25	1.0714 ± 0.0327	0.169					
	2.5	0.1929 ± 0.0107	< 0.001*					
Conbercept	0.01	1.0878 ± 0.0474	0.130					
	0.1	1.1637 ± 0.0421	0.009*					
	1	1.1442 ± 0.0769	0.019*					
5-Fu	0.05	1.0879 ± 0.0353	0.098	Conbercept 0.1/5-Fu	0.05	1.0816 ± 0.0939	0.186	0.892^a^
	0.5	0.8878 ± 0.0329	0.040*		0.5	0.9237 ± 0.0260	0.215	0.092^b^
	5	0.6368 ± 0.0482	< 0.001*		5	0.6508 ± 0.0710	< 0.001*	0.725^ C^
MMC	0.00002	1.0107 ± 0.1753	0.898	Conbercept 0.1/MMC	0.00002	0.9680 ± 0.1601	0.658	0.698^d^
	0.0002	0.8665 ± 0.1158	0.124		0.0002	0.9321 ± 0.0439	0.352	0.270^e^
	0.002	0.6014 ± 0.0558	< 0.001*		0.002	0.5907 ± 0.0465	< 0.001*	0.750^f^

Note: BVZ: bevacizumab; 5-Fu: 5-fluorouracil; MMC: mitomycin C; PBS: Phosphate-buffered saline

the P values: a: 5-Fu 0.05 group vs. Conbercept 0.1 / 5-Fu 0.05 group; b: 5-Fu 0.5 group vs. Conbercept 0.1 / 5-Fu 0.5 group; c: 5-Fu 5 group vs. Conbercept 0.1 / 5-Fu 5 group; d: MMC 0.00002 group vs. Conbercept 0.1 / MMC 0.00002 group; e: MMC 0.0002 group vs. Conbercept 0.1 / MMC 0.0002 group; f: MMC 0.002 group vs. Conbercept 0.1 / MMC 0.002 group

* cell viability of these groups are significantly different from control group (P < 0.05)

**Table 3:** Cell viability of human umbilical vein endothelial cells (HUVECs) after treatment with medicines

Medicine and concentration (mg/ml)		Absorbance at 570 nm (ratio, vs. PBS)	P (vs. control)	Medicine and concentration (mg/ml)		Absorbance at 570 nm (ratio, vs. PBS)	P (vs. control)			P	
Control (PBS)		1.0000 ± 0.0299									
BVZ	0.025	0.8785 ± 0.0590	< 0.001*								
	0.25	0.9029 ± 0.0509	0.003*								
	2.5	1.1203 ± 0.0206	< 0.001*								
Conbercept	0.01	0.8820 ± 0.0224	0.001*								
	0.1	0.9156 ± 0.0539	0.011*								
	1	1.1492 ± 0.0689	< 0.001*								
5-Fu	0.05	0.8064 ± 0.0205	< 0.001*	Conbercept 0.1/5-Fu	0.05	0.7598 ± 0.0407		< 0.001*	0.051^a^		
	0.5	0.6987 ± 0.0127	< 0.001*		0.5	0.7228 ± 0.0225		< 0.001*	0.070^b^		
	5	0.5946 ± 0.0522	< 0.001*		5	0.5637 ± 0.0229		< 0.001*	0.261^c^		
MMC	0.00002	0.8855 ± 0.0212	< 0.001*	Conbercept 0.1/MMC	0.00002	0.8804 ± 0.0156		0.001*	0.680^d^		
	0.0002	0.8869 ± 0.0496	< 0.001*		0.0002	0.8520 ± 0.0537		0.009*	0.317^e^		
	0.002	0.7294 ± 0.0260	< 0.001*		0.002	0.7867 ± 0.0111		< 0.001*	0.003^f^†		

Note: BVZ: bevacizumab; 5-Fu: 5-fluorouracil; MMC: mitomycin C; PBS: Phosphate-buffered saline

the P values: a: 5-Fu 0.05 group vs. Conbercept 0.1 / 5-Fu 0.05 group; b: 5-Fu 0.5 group vs. Conbercept 0.1 / 5-Fu 0.5 group; c: 5-Fu 5 group vs. Conbercept 0.1 / 5-Fu 5 group; d: MMC 0.00002 group vs. Conbercept 0.1 / MMC 0.00002 group; e: MMC 0.0002 group vs. Conbercept 0.1 / MMC 0.0002 group; f: MMC 0.002 group vs. Conbercept 0.1 / MMC 0.002 group

*the cell viability of these groups are significantly different from control group (P < 0.05);

† the cell viability of MMC 0.002 group is significantly different from Conbercept 0.1 / MMC 0.002 group (P < 0.05)

### Drugs inhibited HTF cell migration

**Fig. 2:**
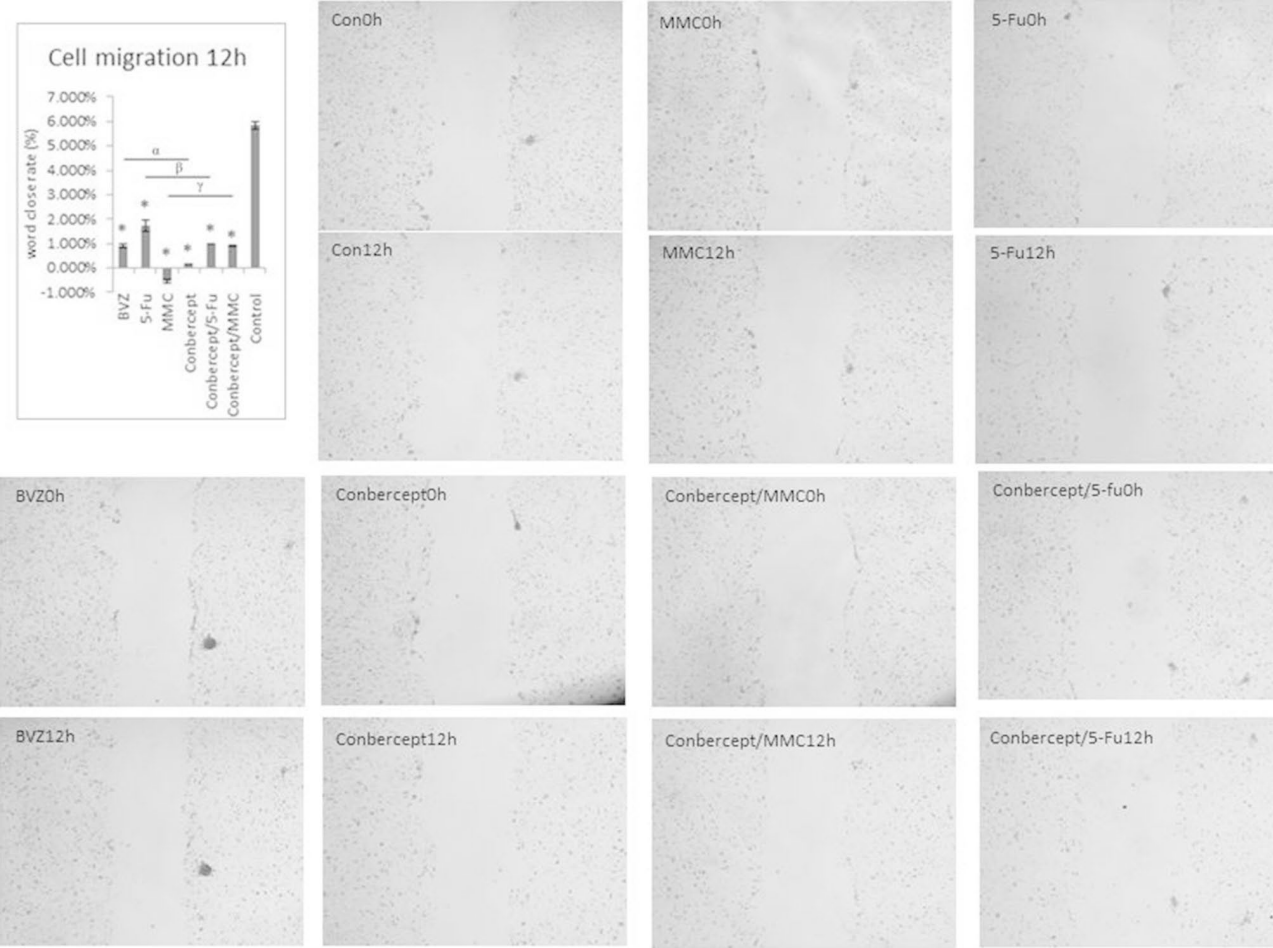
Cell migration assay. The scratch wound assay shows the effects of 5-fluorouracil (5-Fu), mitomycin C (MMC), bevacizumab (BVZ), conbercept, conbercept / 5-Fu, and conbercept / MMC on cell migration of human Tenon’s fibroblasts (HTFs) under the action of VEGF (Magnification, 40×); *P < 0.05

The relative rate of HTF cell migration after 12 h in 5-Fu, MMC, BVZ, conbercept, conbercept + 5-Fu, conbercept + MMC, and control groups was 1.745% ± 0.230%, -0.540% ± -0.093%, 0.915% ± 0.093%, 0.162% ± 0.003%, 0.982% ± 0.019%, 0.900% ± 0.018%, and 5.842% ± 0.154%, respectively, indicating that both drug intervention and combined drug intervention had significant inhibitory effects on HTF cell migration. Conbercept was superior to BVZ and conbercept + 5-Fu was superior to 5-Fu in inhibiting HTF migration (all *P* < 0.05) (Fig. 2).

### Drugs inhibited the expression level of Col1A1 mRNA in HTFs

**Fig. 3:**
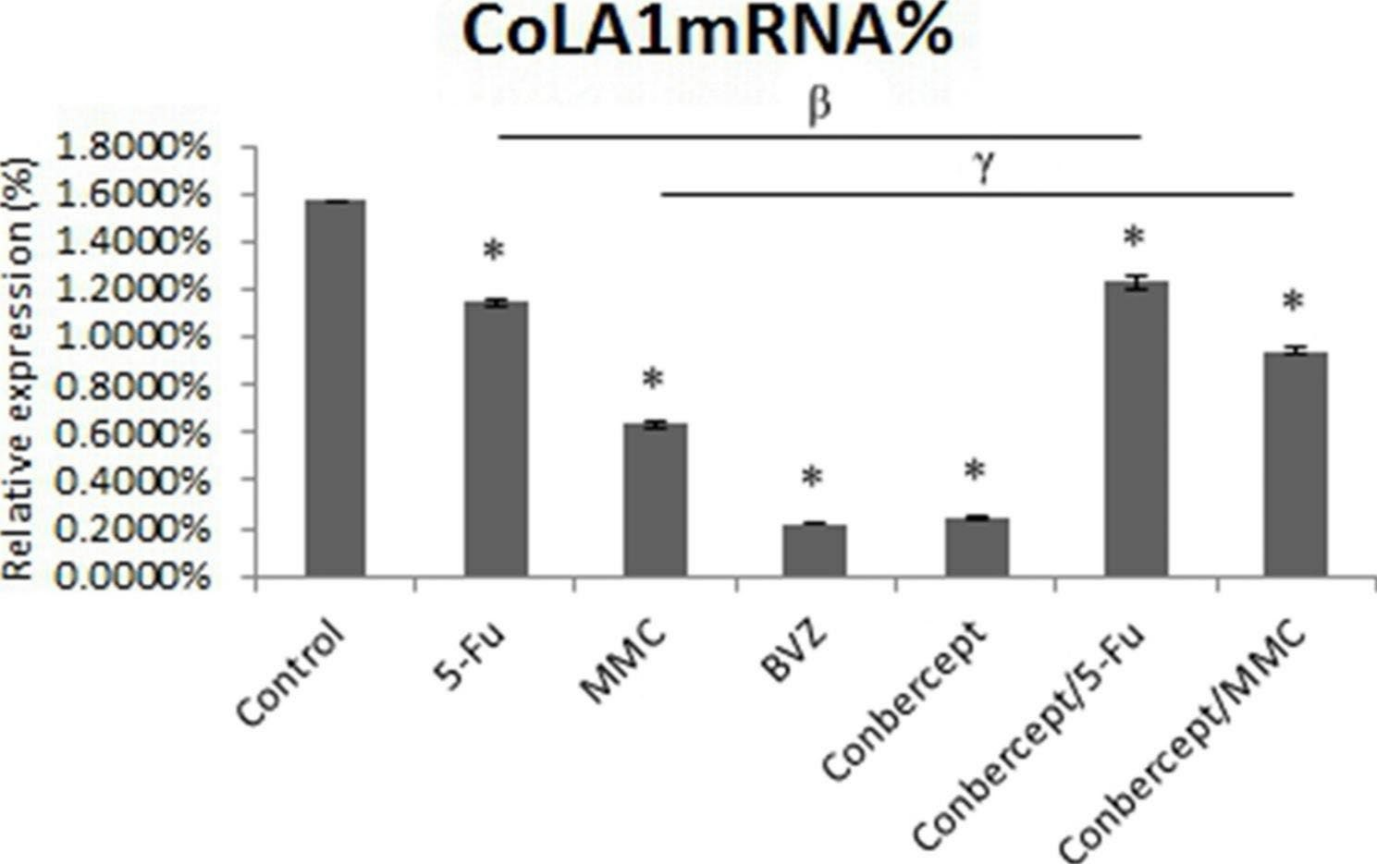
The effects of 5-fluorouracil (5-Fu), mitomycin C (MMC), bevacizumab (BVZ), conbercept, conbecept / 5-Fu, and conbecept / MMC on the expression level of type I collagen alpha 1 (Col1A1) mRNA in human Tenon’s fibroblasts (HTFs); *P < 0.05

The expression level of Col1A1 mRNA in HTFs cultured for 24 h in the control group and 5-Fu, MMC, BVZ, conbercept, conbercept + 5-Fu, and conbercept + MMC groups was 1.5702% ± 0.0051%, 1.1470% ± 0.0111%, 0.6340% ± 0.0098%, 0.2220% ± 0.0061%, 0.2442% ± 0.0045%, 1.2342% ± 0.0273%, and 0.9439% ± 0.0091%, respectively. Drug intervention and combined drug intervention both significantly inhibited the expression level of Col1A1 mRNA in HTFs (all *P* < 0.05) (Fig. 3).

### Inhibitory effects of drugs on the expression level of VEGF(R) mRNA in HTFs

**Fig. 4:**
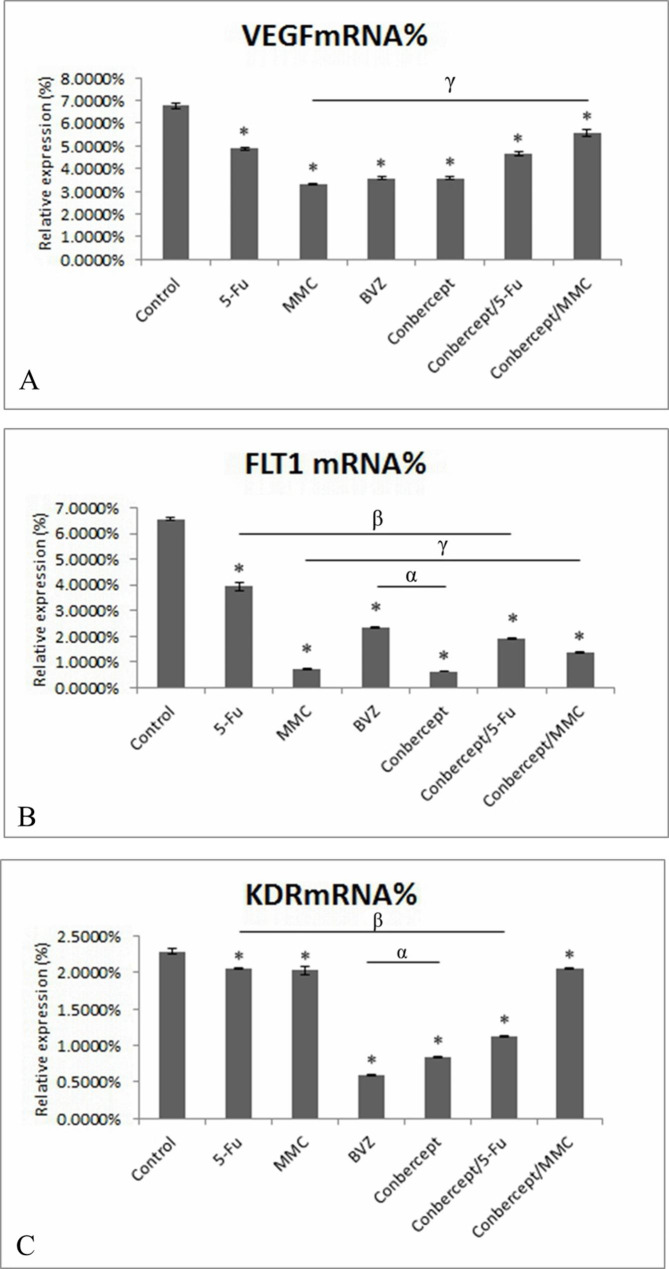
The effects of 5-fluorouracil (5-Fu), mitomycin C (MMC), bevacizumab (BVZ), conbercept, conbecept / 5-Fu, and conbecept / MMC on the expression levels of vascular endothelial growth factor (VEGF), VEGFR-1 (FLT-1), and VEGFR-2 (KDR) mRNA in human Tenon fibroblasts (HTFs); (A) VEGF mRNA%. (B) Flt-1 mRNA%. (C) KDR mRNA%. *P < 0.05

The expression level of VEGF mRNA in HTFs cultured for 24 h in the control group and 5-Fu, MMC, BVZ, conbercept, conbercept + 5-Fu, and conbercept + MMC groups was 6.7691% ± 0.1345%, 4.8778% ± 0.0524%, 3.3322% ± 0.0218%, 3.5661% ± 0.0538%, 3.5745% ± 0.0722%, 4.6492% ± 0.0751%, and 5.5658% ± 0.1360%, respectively; the expression level of VEGFR-1 mRNA in the aforementioned groups was 6.5657% ± 0.0418%, 3.9478% ± 0.1613%, 0.7347% ± 0.0078%, 2.3340% ± 0.0209%, 0.6293% ± 0.0042%, 1.9256% ± 0.0268%, and 1.3669% ± 0.0446%, respectively; and the expression level of VEGFR-2 mRNA in the aforementioned groups was 2.2895% ± 0.0330%, 2.0605% ± 0.0070%, 2.0284% ± 0.0567%, 0.5916% ± 0.0063%, 0.8408% ± 0.0111%, 1.1212% ± 0.0034%, and 2.0484% ± 0.0099%, respectively. Drug intervention and combined drug intervention both significantly downregulated the expression level of VEGF(R) mRNA in HTFs. The effect of conbercept was greater than that of BVZ in inhibiting the expression level of VEGFR-1 mRNA in HTFs. Also, BVZ had a more noticeable effect than conbercept in inhibiting the expression level of VEGFR-2 mRNA in HTFs (all *P* < 0.05) (Fig. 4).

### Suppression of the expression levels of VEGF and PIGF in HUVECs by drugs

**Fig. 5:**
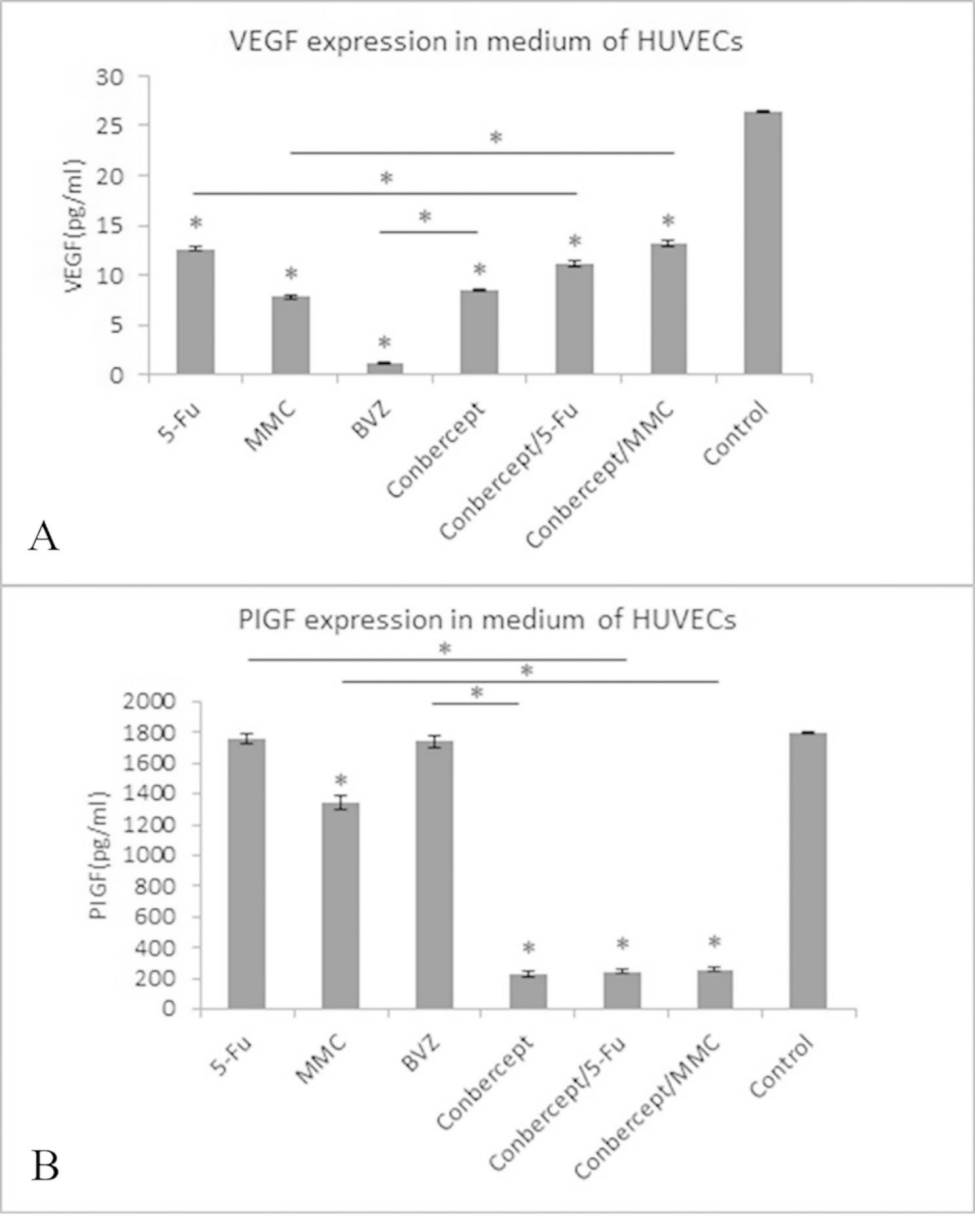
The effects of conbercept, 5-fluorouracil (5-Fu), mitomycin C (MMC), bevacizumab (BVZ), conbercept / 5-Fu, and conbercept / MMC on the expression levels of (A) vascular endothelial growth factor (VEGF) and (B) placental growth factor (PIGF) in human umbilical vein endothelial cells (HUVECs); *P < 0.05

After 24-h culture of HUVECs, the expression level of VEGF in 5-Fu, MMC, BVZ, conbercept, conbercept + 5-Fu, and conbercept + MMC groups (12.6 ± 0.21, 7.77 ± 0.23, 1.15 ± 0.11, 8.47 ± 0.12, 11.1 ± 0.26, and 13.2 ± 0.28 pg/ml) was significantly lower than that in the control group (26.5 ± 0.12pg/ml). Besides, the expression level of VEGF in the BVZ group significantly decreased, which was significantly lower than that in the conbercept group and other groups (all *P* < 0.05). (Fig. 5A)

When HUVECs were cultured and treated with 5-Fu, MMC, BVZ, conbercept, conbercept + 5-Fu, and conbercept + MMC for 24 h, the PIGF level was 1760 ± 34.14, 1340 ± 46.23, 1740 ± 33.76, 230 ± 20.7, 247 ± 12.97, and 260 ± 17.16 pg/ml, respectively. The PIGF levels in HUVECs in the conbercept, conbercept + 5-Fu, conbercept + MMC, and MMC groups were significantly lower than those in the control group (1800 ± 5.04 pg/ml) (all *P* < 0.05) (Fig. 5B).

## Discussion

After GFS, increased angiogenesis in conjunctiva and fibroblast migration at the site of the filtering bleb, leading to fibroblast proliferation with collagen deposition, are the direct causes of filtering bleb failure [5]. Various anti-scarring treatments are adjunctively used for GFS to improve the success rate of surgery. Conbercept has been used as an adjunct in GFS for treating open-angle glaucoma and has been effective in improving the surgical outcome [21]. However, its direct effect on HTF is still unknown, and its mechanism for improving the prognosis of GFS has not been clearly explained. In this study, HTFs were incubated with conbercept, and the direct inhibitory effects of conbercept on HTF cell migration, Col1A1 mRNA expression of HTFs, and VEGF(R) mRNA expression of HTFs were detected. Also, the low cytotoxicity of conbercept was assessed, while the inhibitory effect of concepcept on the expression of PIGF and VEGF in HUVECs was examined.

Clinically, increased bleb vascularity is associated with a poorer prognosis for GFS [30]. VEGF expression increased in the Tenon tissue of patients who experienced failed GFS compared with patients in whom the surgery was successful and patients without glaucoma [31]. VEGF [9, 32] is a key mediator of angiogenesis; inhibiting the VEGF pathway inhibits the angiogenic process [10, 32, 33]. These findings suggest the potential usefulness of anti-VEGF therapy in promoting the success of GFS. Vandewalle et al. [34] and Grewal et al. [35] reported that using BVZ as an adjuvant for GFS could help control IOP after the surgery. However, several anti-VEGF compounds lack efficacy in preventing fibrosis, possibly because PIGF is simultaneously upregulated following the use of anti-VEGF(R) antibodies, leading to a profibrotic effect of PIGF via binding to VEGFR-1 [36]. This leads to an overall profibrotic effect [37].

PIGF is another member of the VEGF family, which shows no effect under physiological conditions, while it is important for pathological angiogenesis, plasma extravasation, and compensatory growth in response to hypoxia, inflammation, wound healing, and cancer [38–40]. Additionally, PIGF is considered as a profibrotic growth factor [41]. Anti-PIGF agents have a direct inhibitory effect on reactive gliosis in the retina [37]. Van Bergen et al. [42] found that the expression level of PIGF in the aqueous humor of patients with glaucoma after anti-VEGF treatment significantly increased, indicating an important contribution of this growth factor to wound healing after trabeculectomy. PIGF can be a possible target for improving the outcome of GFS. Anti-PIGF agents can significantly reduce postoperative proliferation, inflammation, and angiogenesis, as well as collagen deposition in later stage of GFS in animal models [42].

However, treatment with a single antiangiogenic drug may lead to the upregulation of other growth factors. This is based on escape mechanisms via induction of an angiogenic rescue program [42]. Therefore, the combination of anti-VEGF and anti-PIGF agents may attenuate the escape mechanism and affect the three most important wound healing phases: inflammation, angiogenesis, and collagen deposition [42]. In the clinical treatment of vitreoretinal diseases, aflibercept exhibits ambivalent profibrotic effects because it possesses both anti-fibrotic (via PIGF inhibition) and profibrotic properties. After the treatment, the decreased VEGF expression level increases the connective tissue growth factor (CTGF)/VEGF ratio [43, 44], resulting in an overall profibrotic effect [45]. In the choroidal neovascularization model and the mouse streptozotocin model [37], whether the reduction of scar formation after treatment with anti-PIGF antibody is associated with the absence of an angiofibrotic switch (i.e., CTGF release) remains unclear. Nevertheless, it is concluded that the inhibition of PlGF can reduce the process of fibrosis, known as a common side effect of VEGF inhibition [37]. For the anti-scarring effect of GFS, it has been suggested that the optimal dose of anti-PIGF agent combined with the suboptimal dose of anti-VEGF agent (which has no side effects) may better inhibit scarring compared with monotherapy of either [42].

Conbercept has a dual effect on binding to PIGF and VEGF [18–20]. In the present study, conbercept showed a significant inhibitory effect on the PIGF expression with a weaker anti-VEGF effect than BVZ in vascular endothelial cells. It could directly inhibit HTF migration and Col1A1 mRNA expression level in HTFs. Besides, it was found that the inhibitory effect of conbercept on the expression level of VEGFR-1 mRNA in HTFs was more noticeable than that of BVZ. In contrast, the inhibitory effect of conbercept on the expression level of VEGFR-2 mRNA was lower than that of BVZ. It was suggested that conbercept could inhibit the upregulation of PIGF while inhibiting VEGF and also inhibit the signaling pathway of the binding of PIGF to VEGFR-1. Therefore, our study initially indicated that concepcept might be a valuable anti-scarring therapy for GFS. It provided an experimental basis for the clinical application of conbercept as an adjunct in GFS [21].

The results of this study revealed that conbercept + 5-Fu and conbercept + MMC had a remarkable anti-PIGF effect and inferior anti-VEGF effect. Conbercept combined with 5-Fu or MMC could also significantly inhibit HTF migration and the expression of Col1A1 mRNA in HTFs. Conbercept + 5-Fu was superior to 5-Fu in inhibiting HTF migration and expression of VEGF-R1 mRNA and VEGF-R2 mRNA. The cytotoxicity of conbercept combined with 5-Fu or MMC was not higher than that of 5-Fu or MMC, while the cytotoxicity of 2.5 mg/mL BVZ on HTFs was obvious [46]. As a result, the experimental results suggested that the combined use of conbercept with 5-Fu or MMC, especially the combination of conbercept and 5-Fu, might also be effective in delaying the wound healing of GFS.

## Conclusions

The present study showed the direct inhibitory effects of conbercept on HTF migration and Col1A1 mRNA expression level in HTFs, the obvious anti-PIGF and inferior anti-VEGF effects of conbercept compared with BVZ, and the low cytotoxicity of conbercept. In addition, the inhibitory effect of conbercept on VEGFR-1 mRNA expression in HTFs was more pronounced than that of BVZ, and its effect on inhibiting VEGFR-2 mRNA expression was weaker than that of BVZ. This study provided a better understanding of the role of conbercept in the GFS wound healing process.

## Data Availability

All the data supporting the findings of this study are available within the article.

## Abbreviations

BVZ: Bevacizumab
Col1A1: collagen type I alpha1
CTGF: connective tissue growth factor;
DMEM: Dulbecco’s modified Eagle’s medium;
ELISA: enzyme-linked immunosorbent assay;
FBS: fetal bovine serum;
5-Fu: 5-fluorouracil;
GAPDH: glyceraldehyde-3-phosphate dehydrogenase;
GFS: glaucoma filtration surgery;
HUVEC: human umbilical vein endothelial cell;
HTF: human Tenon fibroblast;
IOP: intraocular pressure;
MMC: mitomycin-C;
MTT: 3-(4,5-dimethylthiazol-2-yl)-2,5-diphenyltetrazolium bromide;
PBS: phosphate-buffered saline;
PIGF: placental growth factor;
qPCR: quantitative polymerase chain reaction;
VEGF: vascular endothelial growth factor.
